# Increasing Colorectal Cancer Screening Among Black Men in Virginia: Development of an mHealth Intervention

**DOI:** 10.2196/50028

**Published:** 2024-10-10

**Authors:** Maria D Thomson, Guleer H Shahab, Chelsey A Cooper-McGill, Vanessa B Sheppard, Sherrick S Hill, Michael Preston, Larry Keen II

**Affiliations:** 1 Department of Social and Behavioral Sciences Virginia Commonwealth University Richmond, VA United States; 2 Massey Comprehensive Cancer Center Richmond, VA United States; 3 Department of Health Policy Virginia Commonwealth University Richmond, VA United States; 4 Psychology Department Virginia State University Petersburg, VA United States; 5 Purdue University West Lafayette, IN United States

**Keywords:** colorectal cancer, cancer screening, mHealth, screening, Black men, early detection, health disparities, design thinking

## Abstract

**Background:**

In the United States, colorectal cancer (CRC) is the third leading cause of cancer death among Black men. Compared to men of all other races or ethnicities, Black men have the lowest rates of CRC screening participation, which contributes to later-stage diagnoses and greater mortality. Despite CRC screening being a critical component of early detection and increased survival, few interventions have been tailored for Black men.

**Objective:**

This study aims to report on the multistep process used to translate formative research including prior experiences implementing a national CRC education program, community advisory, and preliminary survey results into a culturally tailored mobile health (mHealth) intervention.

**Methods:**

A theoretically and empirically informed translational science public health intervention was developed using the Behavioral Design Thinking approach. Data to inform how content should be tailored were collected from the empirical literature and a community advisory board of Black men (n=7) and reinforced by the preliminary results of 98 survey respondents.

**Results:**

A community advisory board identified changes for delivery that were private, self-paced, and easily accessible and content that addressed medical mistrust, access delays for referrals and appointments, lack of local information, misinformation, and the role of families. Empirical literature and survey results identified the need for local health clinic involvement as critical to screening uptake, leading to a partnership with local Federally Qualified Health Centers to connect participants directly to clinical care. Men surveyed (n=98) who live or work in the study area were an average of 59 (SD 7.9) years old and held high levels of mistrust of health care institutions. In the last 12 months, 25% (24/98) of them did not see a doctor and 16.3% (16/98) of them did not have a regular doctor. Regarding CRC, 27% (26/98) and 38% (37/98) of them had never had a colonoscopy or blood stool test, respectively.

**Conclusions:**

Working with a third-party developer, a prototype mHealth app that is downloadable, optimized for iPhone and Android users, and uses familiar sharing, video, and text messaging modalities was created. Guided by our results, we created 4 short videos (1:30-2 min) including a survivor vignette, animated videos about CRC and the type of screening tests, and a message from a community clinic partner. Men also receive tailored feedback and direct navigation to local Federally Qualified Health Center partners including via school-based family clinics. These content and delivery elements of the mHealth intervention were the direct result of the multipronged, theoretically informed approach to translate an existing but generalized CRC knowledge–based intervention into a digital, self-paced, tailored intervention with links to local community clinics.

**Trial Registration:**

ClinicalTrials.gov NCT05980182; https://clinicaltrials.gov/study/NCT05980182

## Introduction

Colorectal cancer (CRC) is one of the few cancers for which precancerous and early-stage disease can be identified and treated successfully through regular screening [1,2]. Among African American or Black (Black) men, CRC is the third leading cause of death [3]. Compared to White men, Black men have higher incidence rates (42.3 vs 50.4 per 100,000) and have the highest mortality rate compared to all races and ethnicities (22.3 vs 15.7 per 100,000) [4]. Screening rates among Black men are also between 10% and 30% lower than other racial or ethnic groups, putting them at greater risk for late-stage diagnoses and poorer outcomes [3,5]. Increasing screening among Black men has been a recommended area of focus by the Blue Ribbon Panel for the Cancer Moonshot and endorsed by the National Cancer Advisory Board since the 1970s. Nevertheless, of the 22 evidence-based CRC programs endorsed by the National Cancer Institute (NCI), none are specifically designed for Black men [3,5]. Interventions designed to reach and engage Black men are critical but are not widely available [6-8].

The Screen 2 Save program (S2S) implemented in 2016 was a Center of Health Disparities, NCI-supported collaboration of Community Health Educators in National Outreach Networks and Comprehensive Partnerships to Advance Cancer Health Equity [9,10]. S2S successfully increased CRC knowledge, positively shifted intentions to screen among participants, and was successful in reaching diverse communities across the United States [9]. As one of the national S2S sites, our team successfully enrolled over 233 individuals. Overwhelmingly, participants in our program were women, and only 10% identified as Black men. Others have also identified the need to more effectively reach Black men [8,11,12].

Translational research in cancer risk reduction is described as a multistep process that transforms community observations into “interventions that improve the health of individuals and the public” [13]. Guided by this definition, we report on our multistep process to translate our experiences with S2S into a culturally tailored mobile health (mHealth) intervention. This intervention is designed to increase CRC screening among Black men in Virginia City with one of the highest CRC incidence and mortality rates in the United States. The catchment area served by our institution has been recognized as a national CRC “hot spot” with incidence (45 vs 39 per 100,000) and mortality (16 vs 14 per 100,000) rates that are higher than the US national incidence and mortality rates [1,14], making this a critical gap in CRC screening outreach and engagement in Virginia. To our knowledge, this will be among the first to transform the standardized content of S2S and align the delivery modality with guidance from Black men. This intervention is designed to increase the completion of CRC screening (any test) by Black men at 3 months using a scalable, community-implemented, and easily sharable mHealth platform. This translational science public health intervention is theoretically and empirically informed by our and others’ NCI S2S implementation results and is informed by our community and clinic partners. The following details the design process and implementation plan including a description of milestones in the development process, relationship development with community and clinical partners, and formative data used to develop intervention content, implementation, and evaluation.

## Methods

### Theoretical Model

The Behavioral Design Thinking approach [15] was used to guide the planning, design, and development of the interventional mHealth tool and will guide the implementation and evaluation stages. The Behavioral Design Thinking approach is a framework for developing digital behavior change interventions that merges best practices from 2 key scientific literature: behavioral design (BD) and design thinking (DT). Specifically, methods from these 2 scientific areas are used to guide the identification, development, and testing of content and delivery methods including the Behavioral Change Wheel and APEASE (Acceptability, Practicability, Effectiveness, Affordability, Side-effects, Equity) criteria [16] and Social Cognitive Theory [17,18]. The framework follows five general steps that we have used to organize and describe our translational process and will be noted throughout: (1) empathize with users and their behavior change needs, (2) define user requirements and behavior change requirements, (3) ideate user-centered features and behavior change content, (4) prototype a user-centered solution that supports behavior change, and (5) test against user needs and for behavior change potential [15]. Incorporating this framework to integrate BD and DT throughout the mHealth design process enables more effective and intentional engagement with users, particularly those who are harder to reach including reaching Black men in the community (eg, outside of the medical clinic) about CRC screening.

### Study Design Behavioral Design Thinking Step 1: Empathize With Users and Their Behavior Change Needs

#### Overview

To develop a comprehensive understanding of CRC screening among Black men in Petersburg City and surrounding counties, we began with a multipronged needs assessment using a comprehensive literature review, convening of a community advisory board (CAB) and development of a survey for deployment in our target neighborhoods.

#### CAB Recruitment

A CAB was established to gain members’ perspectives on key barriers to CRC screening and to identify avenues for tailoring the intervention to increase participation among the Black male community around the Petersburg, VA area. CAB members provide input on content development, methods of delivery, and avenues for recruitment. Potential members were identified through existing community partners of Virginia State University and through the VCU Health or Massey Comprehensive Cancer Center Community Outreach and Engagement Office, which has a long history of partnership in the Petersburg area. Letters of invitation were sent electronically and followed up by telephone. Those who were interested were invited to participate in quarterly sessions to provide feedback on content. The initial CAB meeting provided background information on CRC both nationally and locally and then explored CAB members’ attitudes, beliefs, and opinions on what was needed to effectively reach Black men in their city. To do this, the study team presented the existing National S2S standardized educational messaging; CAB members provided critiques and suggestions for tailoring. The focus group was moderated by 2 Black male members of the study team. A semistructured interview guide was used to guide the conversation, and the meeting was audio recorded and transcribed verbatim. Subsequent CAB meetings are held quarterly on Zoom (Zoom Video Communications), lasted approximately 2 hours, and are used to provide project updates and solicit feedback. The CAB consists of 6 male, Black community leaders in Petersburg City and surrounding counties. CAB members represent faith-based communities, barber shops, local businesses, nonprofit organizations, and local government.

#### Survey Development

In addition to the CAB, we launched a survey to collect additional information about potential barriers and facilitators for CRC screening relevant to the tailored intervention. Our goal for the survey was two-fold, we sought to (1) collect firsthand information to examine the interplay between factors identified as important by our CAB including medical mistrust, CRC risk perceptions, health literacy, and physician recommendation and (2) add to the small number of studies that have explicitly examined factors associated with Black men’s participation in CRC screening by examining factors such as racial discrimination [19], masculinity [20], social support [21], and substance use [22,23]. For this paper, we only report on the former as these were used to guide the development of intervention protocols. Health literacy was measured using a 1-item classification that has been used extensively [24]; the lifetime risk of developing CRC was asked using a 5-point Likert response (very likely-very unlikely) adapted from the National Institutes of Health/Health Information National Trends Survey [25]; CRC screening status and barriers were measured using questions adapted from the National Behavioral Risk Factor Surveillance System [26]. Mistrust of health care institutions was measured using the Medical Mistrust Index, which uses a 5-point Likert scale to assess agreement [27].

#### Survey Recruitment

The survey is a self-administered, electronic survey that takes 20-30 minutes to complete. It is housed and delivered using a HIPAA (Health Insurance Portability and Accountability Act)-compliant REDCap (Research Electronic Data Capture; Vanderbilt University) server. Using a QR code, participants access a secure, unique link and complete a screener to determine eligibility, a self-administered study information form with consent elements followed by the survey. Eligible participants self-identify as Black or African American, are aged 45-75 years, living or working in counties of interest, and have no personal history of cancer. Recruitment is conducted through social media, printed postcards, and posters displayed throughout businesses in the community, and through presentations by study staff at community events.

#### Developing Partnerships With Community Clinics

To be responsive to the empirical literature, our institutional implementation of S2S results and CAB recommendations, partnerships were sought with local Federally Qualified Health Center (FQHC) clinics. These partnerships are critical to address 2 components of the intervention. First, with clinic partners, we can have unique recruitment opportunities. A total of 2 of the 4 clinics operated by our FQHC partner are embedded in local schools, which will be key for reaching adult family members of students. CAB members identified the importance of family in reaching men and prompting screening. This also aligns with the current goal of our clinic partners and their community health educators who are focusing on CRC education that is delivered to families within the school clinic environment. Second, clinic partners are needed to provide direct access to obtaining a CRC screen. All men will also be asked if they would prefer having a clinic member contact them to set up an appointment with either the Massey Cancer Center navigation team or the local FQHC.

### Ethical Considerations

This project underwent a human participant research ethics review by the Virginia Commonwealth University Institutional Review Board and was classified as exempt status (HM20023619). Participants are provided a self-administered research letter that explains all required elements of consent. Study data are stored, analyzed, and published in a deidentified format. CAB members received US $50 per meeting attended; survey respondents received US $20.

## Results

### Behavioral Design Thinking Step 2: Define User and Behavior Change Requirements

#### Literature Review Key Findings That Informed Intervention Design

Interventions that showed significant increases in CRC screening among Black men incorporated components of patient navigation and increasing access via free immunologic fecal occult blood tests [28]. Connecting patients to FQHCs serving individuals who are medically underserved and uninsured was also identified as successful in increasing rates of screening completion [29].

Individual-level factors associated with lower completion rates of CRC screening include lack of physician recommendation, medical mistrust, perceived discrimination, and avoidance [5,6,30-35]. For Black men, avoidance, fear, and perceived discrimination contribute to low screening and play a critical role in their health care use, health behaviors, and mortality [5,30,33,34,36]. Mistrust of the health care system and experiences of racial discrimination are associated with lower health services use and are widely cited as attitudinal barriers to CRC screening [36-38]. Black men expressed explicit fears of medical experimentation and uneasiness about the invasiveness of CRC screening procedures (eg, colonoscopy) [28,37,39-41].

Social support was correlated to receipt of a colonoscopy [41-43]. Kinney et al [42] found that the association between social connection and CRC screening was stronger for Black Americans than for White Americans. Supportive social networks (eg, friends and community leaders) endorsed and encouraged the prioritization of personal preventive health behaviors while simultaneously attenuating fear and embarrassment that impede screening uptake [44].

#### Synthesis of CAB Recommendations

In partnership with our CAB members, 4 barriers to CRC screening were identified that were considered key to tailoring for our target community. Mistrust in the medical system came up numerous times, specifically the perception that clinicians do not fully inform patients of the need for CRC screening routinely beginning at age 45 years. CAB members discussed their own and others’ experiences of having to ask clinicians about CRC screening rather than having it offered or raised as a component of preventive care. For some, the time demands for obtaining a referral, attending a colonoscopy appointment, and arranging for transportation afterward were seen as critical access barrier. Similarly, not having a regular or local source of care was also mentioned. For all of these reasons, the CAB members felt it was critical to have a way of asking men whether they wished to learn more and participate in CRC screening and have the intervention provide a direct link to local, easily accessible clinics capable of providing different types of CRC screening (eg, fecal immunochemical test vs colonoscopy). The lack of information and misinformation about screening test options was also discussed and CAB members expressed concern that information needed to be presented that specifically related to CRC risks for Black men and described the different test options so that there was greater awareness and diminished misinformation. Finally, families, particularly women and children, were noted as highly influential to the choice of whether to participate in screening. Men discussed wanting to complete screening to ensure that they stay healthy and remain able to care for family members and fulfill their familial role. Additionally, women were noted as being a key source of support and encouragement for men to prioritize their preventive health care. These were noted as key aspects of outreach and recruitment. It was noted that intervention reach would likely be much greater if men, women, and children were each targeted; the idea was that women and children could successfully introduce the topic and encourage participation.

#### Preliminary Survey Results

##### Lessons Learned for the Intervention Launch

To date, 126 responses have met inclusion criteria and 79.4% (100) of them have consented; a total of 98 participants have completed the survey. No data are collected from those who do not consent. The mean age of participants was 59 (SD 7.9) years; 29.6% (29/98) of them were covered by Medicaid, 30.6% (30/98) by Medicare, and 8% (8/98) of them were uninsured. In the last 12 months, 25% (24/98) of them had not seen a doctor, 17% (17/98) of them did not have a regular doctor, and 16% (16/98) of them were comfortable filling out medical forms, an indicator of health literacy. Regarding CRC screening, 26% (25/98) of them and 39% (38/98) of them had never had a colonoscopy or blood stool test, respectively. Mistrust of health care organizations is displayed in Table 1; our results show moderate to high levels of agreement with all 7 statements, indicating a generally elevated level of mistrust of health care organizations. These data support the intervention focus on trust and direct linkages to clinics within the community to support access to CRC screening.

**Table 1:** Medical mistrust of health care organizations.

Characteristics	Strongly agree or agree, n	Neutral, n	Strongly disagree or disagree, n
Mistakes are common in health care organizations	36	26	23
Wonder if they really know what they are doing	44	21	17
Health care organizations do not always keep your information private	46	19	18
Health care organizations have sometimes done harmful experiments	56	15	14
When health care organizations make mistakes they usually cover them up	46	24	13
Patients have sometimes been deceived or mislead by health care organizations	53	16	13
You better be cautious when dealing with health care organizations	58	13	14

### Behavioral Design Thinking Step 3: Ideate User-Centered Features and Behavior Change Content

**Figure 1:**
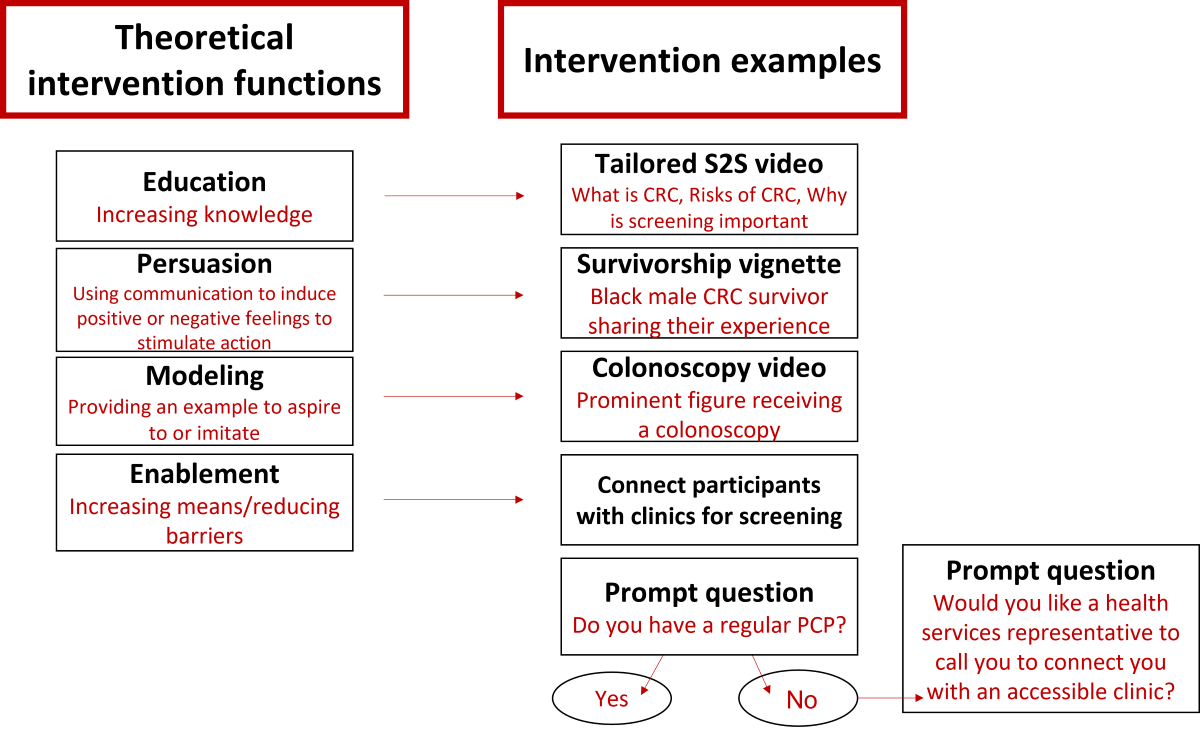
Identified constructs to be addressed and the aligning intervention component. CRC: colorectal cancer; PCP: primary care provider; S2S: Screen 2 Save.

Key user features that we have integrated as a direct result of the literature review and CAB suggestions include opportunities for sharing information with social networks, opportunities for men to request clinic appointments, content explaining types of screening tests, and testimonials from Black men about their experiences with these tests. Moreover, our work is aligned with current NCI roundtable goals of addressing screening rates among communities at highest risk using evidence-based, cost-effective, and culture-specific techniques. The schematic in Figure 1 illustrates our data mapping process that was used to align results from the literature review and CAB findings to the Michie Behavior Change Wheel [16] and the subsequent identification of corresponding intervention functions including education, persuasion, modeling, and enablement. Moreover, our preliminary survey results support several of the areas identified by the CAB as key factors to be included in the intervention such as the need for clinic partnerships to support access for those who do not have a regular physician or insurance and the need to address medical mistrust.

### Behavioral Design Thinking Step 4 (Prototype): A User-Centered Solution That Supports Behavior Change

#### Overview

Working with Pattern Health, a for-profit digital health platform developer [45], we developed a prototype mHealth app to engage participants and deliver content in a way that enables them to choose which aspects to view and share with their social network, receive tailored messages that encourage CRC screening, and provides a referral to a local clinic for CRC screening. The app is downloadable, optimized for iPhone and Android users, and uses familiar common sharing, video playing, and SMS text messaging modalities. Men will be asked to download the app at no cost from their usual app store. Once downloaded, they will complete a short, self-directed screener to assess study eligibility and complete the consent form and baseline measures. They will then gain access to all content, including short videos, tailored feedback, and navigation, to local clinics. All videos and infographics will be sharable using common social media. The following sections describe the specific components developed.

#### Short Videos

To address education and persuasion, the S2S standardized education was transformed into 2 short animated videos tailored for Black men. These covered a basic description of CRC, modifiable risk factors, and the importance of screening (1:30 minutes). The second video, at 2:30 minutes, explained survival by stage at diagnosis and the different screening options available. To address modeling and persuasion, a third video depicting a CRC survivor detailing the importance of screening, early detection, and dispelling common myths about screening was created. The CAB felt strongly that it was important to include a testimonial from a Black male survivor, also available is a video of a well-known Black male actor obtaining a colonoscopy that was created to encourage greater information sharing and dispelling of misinformation about the screening procedure. Finally, we have a short introductory video created by a community leader who works for a local nonprofit; this video is meant to introduce and orient users to the importance of CRC screening and the mHealth app content.

#### Tailored Feedback

After enrollment, participants who have not interacted with the app content for 1 week will receive a SMS text message asking about their readiness to engage in CRC screening. The 1-item question is based on the stages of change model [46] and is adapted to ask about readiness to engage in CRC screening. Each of the 5 readiness-to-change stages is represented and tailored to ask about CRC screening (precontemplation, contemplation, preparation, action, and maintenance). Messages to each response have been developed and will direct the participant to specific content within the mHealth app. These tailored messages are designed to optimize support for CRC screening by tailoring to select barriers, concerns, and personal values of the participant. For example, men who respond that they are unsure if they will get screened will receive a message directing them to the video of the CRC survivor who discusses why screening is important. We will track who receives the readiness to screen questions, their response, and whether they interact with the recommended content.

#### Navigation to CRC Screening

The mHealth app is designed to engage with care in 2 ways. First, participants will be asked if they have a regular source of care who they are willing to discuss CRC screening with. For those who do, they will be provided a list of questions to help guide their conversations. Those who do not have a regular source of care or who indicate they are not interested in discussing CRC screening with that provider will be asked if they would like someone from the local clinic to contact them. Notification of participant interest will be made directly from the mHealth platform. Regardless of the response, it will be important to understand who does and who does not choose to speak to a known provider or contact the local clinic. Factors underlying these choices will be elicited during the intervention exit interviews.

#### Family History

In our previous work, we and others identified that the family history of CRC is often unknown and undiscussed. Given the CAB members’ emphasis on the importance of family in decision-making, we have included a visually appealing infographic that lists 4 descriptive relationships that enable men to contextualize what is meant by familial risk. Participants can use this to quickly and easily identify the most pertinent information about what is meant by family history (eg, which relationships this refers to) and how can this information be discussed with others in the family. It also describes the most salient information about why knowing your family history is important.

## Discussion

### Principal Findings

Using an implementation science approach to adapt findings from our previous experiences with S2S, we were able to identify a key community of Black men in our cancer center catchment who were underusing CRC screening and build a tailored intervention. Guided by the steps of the BDDS framework, we triangulated data collected from a review of the literature, the CAB, and a survey of Black men living or working in the geographic area of focus. Key topics that were identified as critical for the intervention included tailored information about CRC that was focused on and delivered by Black men, the need for easy-to-navigate and direct linkages to community clinics to support receipt of CRC screening, and the inclusion of family to assist with recruitment and information sharing. Working with a third-party developer, we then developed a prototype mHealth app that is downloadable, optimized for iPhone and Android users, and uses familiar sharing, video, and text messaging modalities.

The popularity and ease of access have positioned digital apps to be used successfully at the population level to deliver cancer risk reduction interventions. Continuing to plague these tools has been difficult with reach and engagement, particularly within diverse communities [47-49]. Many interventions using unique and potentially scalable apps have limited reach or remain untailored and unresponsive to critical community needs [47,49]. Yet, given the increasing ease and timing with which these tools can be modified and updated, tailoring to specific community needs becomes less of a barrier. The approach described here used both tailoring content to the specific population and the integration of known behavior change techniques. It is anticipated that together, this content will both engage men and address the key barriers to CRC screening in a manner that supports and encourages them to get screened. The implementation plan is presented in step 5 (testing against user needs and for behavior change potential), which is outlined in Multimedia Appendix 1 [50] and the initial pilot is currently underway.

### Limitations

Limitations to this work are noted. This is a report on the development of an mHealth app that is undergoing the initial stages of user testing and efficacy assessment. Therefore, the sample sizes are small. However, it has been developed using empirical, theoretical, and community partner input. Limits to generalizability to note are that this mHealth app is designed to engage Black men in a specific geographical area in CRC screening. However, if successful, the model of linking community-specific partners and information with an mHealth delivery still has the potential to be scalable. Community health workers and health promotors could leverage existing partnerships with FQHCs to customize the contact information and touchpoints, but the short videos would need little customization. Additional short video content should also be developed and tested that is tailored to other groups such as Black women, and other race, ethnicity, and language groups. Such tests would require evaluation of how receptive participants are to mHealth delivery of cancer screening information.

### Conclusions

Many screening interventions focus on existing patients who are already associated with primary care. Interventions are also needed that focus on ways to identify individuals who are currently disconnected from regular care and preventive cancer screening and can successfully re-establish or build new linkages between individuals and preventive care such as local community clinics. The ability to bring the information to people who are currently unassociated with health care centers or clinics, in a modality that is private but sharable using familiar social media features, and leverages partnerships with local clinics, may be a successful way of linking community outreach to facilitate CRC screening with clinical care.

## Appendix

Multimedia Appendix 1 Summary of the intervention pilot that will be undertaken as a result of the formative work reported in this paper.

## Abbreviations

APEASE: Acceptability, Practicability, Effectiveness, Affordability, Side-effects, Equity
BD: behavioral design
CAB: community advisory board
CRC: colorectal cancer
DT: design thinking
FQHC: Federally Qualified Health Center
HIPAA: Health Insurance Portability and Accountability Act
mHealth: mobile health
NCI: National Cancer Institute
REDCap: Research Electronic Data Capture
S2S: Screen 2 Save

## Data Availability

The data sets generated and analyzed during this study are available from the corresponding author on reasonable request.

## References

[1] Siegel RL, Sahar L, Robbins A, Jemal A (2015). Where can colorectal cancer screening interventions have the most impact?. Cancer Epidemiol Biomarkers Prev.

[2] Centers for Disease Control Prevention (CDC) (2011). Vital signs: colorectal cancer screening, incidence, and mortality—United States, 2002-2010. MMWR Morb Mortal Wkly Rep.

[3] (2022). American Cancer Society.

[4] Cancer stat facts: colorectal cancer. National Cancer Institute.

[5] Kwaan MR, Jones-Webb R (2018). Colorectal cancer screening in black men: recommendations for best practices. Am J Prev Med.

[6] Winterich JA, Quandt SA, Grzywacz JG, Clark PE, Miller DP, Acuña J, Arcury TA (2008). Masculinity and the body: how African American and White men experience cancer screening exams involving the rectum. Am J Mens Health.

[7] Rogers CR, Okuyemi K, Paskett ED, Thorpe RJ, Rogers TN, Hung M, Zickmund S, Riley C, Fetters MD (2019). Study protocol for developing #cuttingCRC: a barbershop-based trial on masculinity barriers to care and colorectal cancer screening uptake among African-American men using an exploratory sequential mixed-methods design. BMJ Open.

[8] Rogers CR, Matthews P, Xu L, Boucher K, Riley C, Huntington M, Le Duc N, Okuyemi KS, Foster MJ (2020). Interventions for increasing colorectal cancer screening uptake among African-American men: a systematic review and meta-analysis. PLoS One.

[9] Whitaker DE, Snyder FR, San Miguel-Majors SL, Bailey LAO, Springfield SA (2020). Screen to save: results from NCI's colorectal cancer outreach and screening initiative to promote awareness and knowledge of colorectal cancer in racial/ethnic and rural populations. Cancer Epidemiol Biomarkers Prev.

[10] Screen to save: NCI colorectal cancer outreach and screening initiative. National Cancer Institute.

[11] Woods VD, Montgomery SB, Belliard JC, Ramirez-Johnson J, Wilson CM (2004). Culture, black men, and prostate cancer: what is reality?. Cancer Control.

[12] O'Malley A, Beaton E, Yabroff KR, Abramson R, Mandelblatt J (2004). Patient and provider barriers to colorectal cancer screening in the primary care safety-net. Prev Med.

[13] Austin CP (2018). Translating translation. Nat Rev Drug Discov.

[14] Thatcher EJ, Camacho F, Anderson RT, Li L, Cohn WF, DeGuzman PB, Porter KJ, Zoellner JM (2021). Spatial analysis of colorectal cancer outcomes and socioeconomic factors in Virginia. BMC Public Health.

[15] Voorheis P, Zhao A, Kuluski K, Pham Q, Scott T, Sztur P, Khanna N, Ibrahim M, Petch J (2022). Integrating behavioral science and design thinking to develop mobile health interventions: systematic scoping review. JMIR Mhealth Uhealth.

[16] Michie S, van Stralen MM, West R (2011). The behaviour change wheel: a new method for characterising and designing behaviour change interventions. Implement Sci.

[17] Bandura A (2004). Health promotion by social cognitive means. Health Educ Behav.

[18] Bandura A (2022). Self-Efficacy: The Exercise of Control.

[19] Ibekwe LN, Fernández-Esquer ME, Pruitt SL, Ranjit N, Fernández ME (2023). Associations between perceived racial discrimination, racial residential segregation, and cancer screening adherence among low-income African Americans: a multilevel, cross-sectional analysis. Ethn Health.

[20] Carethers JM (2022). Closing the gap: how masculinity affects colorectal cancer screening in African-American men. Dig Dis Sci.

[21] Redwood D, Provost E, Asay E, Ferguson J, Muller J (2013). Giant inflatable colon and community knowledge, intention, and social support for colorectal cancer screening. Prev Chronic Dis.

[22] Amersi F, Agustin M, Ko CY (2005). Colorectal cancer: epidemiology, risk factors, and health services. Clin Colon Rectal Surg.

[23] Goding Sauer A, Siegel RL, Jemal A, Fedewa SA (2019). Current prevalence of major cancer risk factors and screening test use in the United States: disparities by education and race/ethnicity. Cancer Epidemiol Biomarkers Prev.

[24] Chew LD, Griffin JM, Partin MR, Noorbaloochi S, Grill JP, Snyder A, Bradley KA, Nugent SM, Baines AD, Vanryn M (2008). Validation of screening questions for limited health literacy in a large VA outpatient population. J Gen Intern Med.

[25] HINTS 6 survey instrument NCI. National Cancer Institute.

[26] (2019). BRFSS statistical brief on colorectal cancer screening questions. CDC, Division of Cancer Prevention and Control.

[27] LaVeist TA, Nickerson KJ, Bowie JV (2000). Attitudes about racism, medical mistrust, and satisfaction with care among African American and white cardiac patients. Med Care Res Rev.

[28] Basch CH, Basch CE, Zybert P, Wolf RL (2016). Fear as a barrier to asymptomatic colonoscopy screening in an urban minority population with health insurance. J Community Health.

[29] Jaqua E, Nguyen V, Morton K, Chin E, Brougher A, Dawes J (2022). Improving cervical cancer screening rates at an urban federally qualified health center family medicine residency clinic. Perm J.

[30] Cole H, Thompson HS, White M, Browne R, Trinh-Shevrin C, Braithwaite S, Fiscella K, Boutin-Foster C, Ravenell J (2017). Community-based, preclinical patient navigation for colorectal cancer screening among older black men recruited from barbershops: the MISTER B trial. Am J Public Health.

[31] Fyffe DC, Hudson SV, Fagan JK, Brown DR (2008). Knowledge and barriers related to prostate and colorectal cancer prevention in underserved black men. J Natl Med Assoc.

[32] Kang SH, Bloom JR (1993). Social support and cancer screening among older black Americans. J Natl Cancer Inst.

[33] Himmelstein MS, Sanchez DT (2016). Masculinity impediments: internalized masculinity contributes to healthcare avoidance in men and women. J Health Psychol.

[34] Walsh-Childers K, Odedina F, Poitier A, Kaninjing E, Taylor G (2018). Choosing channels, sources, and content for communicating prostate cancer information to black men: a systematic review of the literature. Am J Mens Health.

[35] Rogers CR, Mitchell JA, Franta GJ, Foster MJ, Shires D (2017). Masculinity, racism, social support, and colorectal cancer screening uptake among African American men: a systematic review. Am J Mens Health.

[36] Suite DH, La Bril R, Primm A, Harrison-Ross P (2007). Beyond misdiagnosis, misunderstanding and mistrust: relevance of the historical perspective in the medical and mental health treatment of people of color. J Natl Med Assoc.

[37] Fiscella K, Franks P, Clancy CM (1998). Skepticism toward medical care and health care utilization. Med Care.

[38] Crawley LVM, Ahn DK, Winkleby MA (2008). Perceived medical discrimination and cancer screening behaviors of racial and ethnic minority adults. Cancer Epidemiol Biomarkers Prev.

[39] Honein-AbouHaidar GN, Kastner M, Vuong V, Perrier L, Daly C, Rabeneck L, Straus S, Baxter NN (2016). Systematic review and meta-study synthesis of qualitative studies evaluating facilitators and barriers to participation in colorectal cancer screening. Cancer Epidemiol Biomarkers Prev.

[40] Nagelhout E, Comarell K, Samadder NJ, Wu YP (2017). Barriers to colorectal cancer screening in a racially diverse population served by a safety-net clinic. J Community Health.

[41] Bynum SA, Davis JL, Green BL, Katz RV (2012). Unwillingness to participate in colorectal cancer screening: examining fears, attitudes, and medical mistrust in an ethnically diverse sample of adults 50 years and older. Am J Health Promot.

[42] Kinney AY, Bloor LE, Martin C, Sandler RS (2005). Social ties and colorectal cancer screening among blacks and whites in North Carolina. Cancer Epidemiol Biomarkers Prev.

[43] Myers RE, Sifri R, Hyslop T, Rosenthal M, Vernon SW, Cocroft J, Wolf T, Andrel J, Wender R (2007). A randomized controlled trial of the impact of targeted and tailored interventions on colorectal cancer screening. Cancer.

[44] Lumpkins CY, Vanchy P, Baker TA, Daley C, Ndikum-Moffer F, Greiner KA (2016). Marketing a healthy mind, body, and soul: an analysis of how African American men view the church as a social marketer and health promoter of colorectal cancer risk and prevention. Health Educ Behav.

[45] (2023). Pattern health.

[46] DiClemente CC, Prochaska JO (1998). Toward a comprehensive, transtheoretical model of change: stages of change and addictive behaviors.

[47] King AC, Hekler EB, Grieco LA, Winter SJ, Sheats JL, Buman MP, Banerjee B, Robinson TN, Cirimele J (2016). Effects of three motivationally targeted mobile device applications on initial physical activity and sedentary behavior change in midlife and older adults: a randomized trial. PLoS One.

[48] King AC, Campero MI, Sheats JL, Castro Sweet CM, Hauser ME, Garcia D, Chazaro A, Blanco G, Banda J, Ahn DK, Fernandez J, Bickmore T (2020). Effects of counseling by peer human advisors vs computers to increase walking in underserved populations: the COMPASS randomized clinical trial. JAMA Intern Med.

[49] Hesse BW, Kwasnicka D, Ahern DK (2021). Emerging digital technologies in cancer treatment, prevention, and control. Transl Behav Med.

[50] Lancaster GA, Dodd S, Williamson PR (2004). Design and analysis of pilot studies: recommendations for good practice. J Eval Clin Pract.

