# Supplementary material for: Increasing Colorectal Cancer Screening Among Black Men in Virginia: Development of an mHealth Intervention
Source: JMIR Form Res. 2024 Oct 10;8:e50028. doi: 10.2196/50028 (PMC11502976; doi:10.2196/50028)
Supplement: Multimedia Appendix 1 [file formative_v8i1e50028_app1.docx]

*Step 5 Test against user needs and for behavior change potential.*

A single arm, preliminary test of feasibility and efficacy will be conducted using a sample of n=30 men recruited from the community.[50] The primary efficacy outcome is completion of CRC screening (any test) at 12 weeks as reported by the participants. Feasibility will be assessed using recruitment, engagement and retention benchmarks. Research measures will be collected at baseline, completion of the intervention and 12 weeks post intervention. Risk assessment

*Population and Site*

Eligibility Criteria. Men who identify as Black or African American, aged 45-75 years, eligible for CRC screening (e.g., no FIT in last 12 months OR colonoscopy in last 10 years), no personal cancer diagnosis, live or work in one of the study site counties, and are not a CAB member.

*Recruitment*

One of the unique components of this intervention is the use of a mobile platform to provide a private viewing experience that is easily sharable in contrast to traditional community settings for cancer education that are often group based, public conversations at scheduled dates and locations. This change was deemed critical by the CAB members who emphasized both privacy and the ability to share the information. It was also important that recruitment occur in the community and focus on individuals who may not be associated with a health clinic or regular primary care. To recruit these individuals we leveraged the Massey Community Champions, cancer advocates who live and work in the community. The Cancer Champions are part of a statewide network of individuals who assist and inform research occurring within the cancer center service area. Cancer Champions are trained and supported by Massey Cancer Center in human subjects research, cancer communication, clinical trials and various cancer screening topics. This training combined with their strong presence in their own communities uniquely readies them to recruit successfully. Moreover, recruitment strategies target families in addition to the men themselves, as our CAB members have strongly emphasized the importance of family support and encouragement when deciding to participate in screening.

*Measures*

Research measures collected at baseline, exit and 12 weeks post are: CRC knowledge, risk perception, medical mistrust, preventive health behaviors. At 12 week only, CRC screening or intent to screen, and participant satisfaction with the intervention will be asked. Process evaluation will assess any change in CRC knowledge, risk perception, medical mistrust, and preventive health behaviors at 12 weeks.

Feasibility will be assessed using a priori benchmarks for recruitment and retention. Recruitment success will be represented by achieving 100% of the recruitment goal. Acceptance rates and reasons for declining participation will be tracked. Information on refusals will be used to modify recruitment methods as needed. Retention benchmarks will be supported if >=65% complete both weeks 2 and 12 follow up. Engagement benchmarks will be supported if >=65% of intervention completers who do not have a primary care provider chose to receive navigation services; 80% of those consented view intervention materials (e.g., click on text/graphical data, view videos, complete risk assessment).

Preliminary Efficacy. A signal for efficacy will be considered positive if among those who complete the intervention a) most (≥60%) complete CRC screening and b) >80% acknowledge their intent to get screened.

Follow-up interviews. Interviews will be conducted with all participants to collect in-depth, descriptive feedback on satisfaction with the intervention content, delivery modalities, ease of use and usefulness. We will also interview clinic staff and Community Champions to gain insight into facilitators and barriers to recruitment efforts and response of the community to the intervention. Suggestions for additions and alterations will be solicited from all interviews.
